# Effect of Delta-9-tetrahydrocannabinol and cannabidiol on milk proteins and lipid levels in HC11 cells

**DOI:** 10.1371/journal.pone.0272819

**Published:** 2022-08-17

**Authors:** Chitmandeep Josan, Tina Podinic, Nikko Pfaff, Sandeep Raha

**Affiliations:** Department of Pediatrics and the Graduate Programme in Medical Sciences, McMaster University, Hamilton, Ontario, Canada; Rush University Medical Center, UNITED STATES

## Abstract

Pregnant and lactating women have been discouraged from using cannabis by Health Canada. However, the increasing rate of cannabis use among pregnant women has presented an urgent need to investigate its physiological effects during the perinatal period. During pregnancy, the mammary gland (MG) undergoes remodeling, which involves alveolar differentiation of mammary epithelial cells (MECs), which is essential for breast milk production and secretion. Limited evidence has been reported on the impact of cannabis or its components, delta-9-tetrahydrocannabinol (THC) and cannabidiol (CBD), on MG development or MEC differentiation. In this study, we investigated the effects of THC and CBD on the differentiation of MECs by assessing changes in cellular viability, lipid accumulation, and gene and protein expression of major milk protein and lipid synthesizing markers. using the HC11 cells as a model. We hypothesized that THC and CBD will negatively impact the synthesis of milk proteins and lipids, as well as lipid markers in HC11 cells. Our results demonstrated that THC and CBD reduced cellular viability at concentrations above 30μM and 20μM, respectively. Relative to control, 10μM THC and 10μM CBD reduced mRNA levels of milk proteins *(CSN2* and *WAP)*, lipid synthesizing and glucose transport markers *(GLUT 1*, *HK2*, *FASN*, *FABP4*, *PLIN2* and *LPL)*, as well as whey acidic protein and lipid levels. In addition, co-treatment of a CB2 antagonist with THC, and a CB2 agonist with CBD, reversed the impact of THC and CBD on the mRNA levels of key markers, respectively. In conclusion, 10μM THC and CBD altered the differentiation of HC11 cells, in part via the CB2 receptor.

## Introduction

Cannabis is one of the most common substances used by women during pregnancy for alleviating nausea, vomiting and other symptoms of morning sickness [1,2]. The prevalence of cannabis use has been reported to be high among Canadians of reproductive age, with 44% of individuals aged 16 to 19, 52% aged 20 to 24, and 24% aged 25 years reporting use [3]. Cannabinoids have been reported to cross the placenta [4,5]. The usage of cannabis during pregnancy has been been associated with poor fetal growth [6,7], low birth weight [8–10], smaller head circumference [11], and increased risk of premature birth [9]. These associations have led the Society of Obstetricians and Gynaecologists of Canada to recommend that women should discontinue the use of cannabis during pregnancy [12]. Despite the recommendation, cannabis use by women during pregnancy has been reported to be between 2 and 11.3% [8,13–18], with prevalence among low-income and disadvantaged pregnant women ranging between 14 and 28% [19–21]. Taken together, the high rate of cannabis use among Canadians of reproductive age [3,22] and use during pregnancy has created an urgent need to understand its impact on female reproductive organs and infant health.

Cannabis contains over 400 chemical components, including the psychoactive component delta-9-tetrahydrocannabinol (THC) and the antiemetic component cannabidiol (CBD) [23]. THC and CBD, bind to and differentially activate cannabinoid receptor 1 (CB1) and CB2 [24], which are key receptors of the endocannabinoid system (ECS). In addition to modulating pain and neurodevelopment, the ECS has been reported to impact implantation and placentation [25]. Dysregulation of ECS signaling by THC and CBD may impact critical processes, resulting in altered cell proliferation, differentiation and function [25,26]. THC treatment has been found to reduce proliferation and invasion of human extravillous trophoblast cells [27], which are outcomes linked to poor placentation [28]. In trophoblasts cells, CBD treatment caused loss of cell viability, disruption in cell cycle progression and apotosis through a mitochondrial pathway [29]. Altogether, both THC and CBD have been demonstrated to impact cellular differentiation and function, however their impact on the differentiation of mammary epithelial cells (MECs) remains unclear.

Alveolar differentiation of MECs takes place within the mammary gland (MG) during pregnancy to prepare the gland for milk production [30]. Differentiated MECs are capable of synthesizing and secreting lactose [31], fat [32], and milk proteins, such as β-casein and whey acidic protein (WAP) [33–35]. Improper differentiation of secretory MECs is associated with fewer alveoli, decreased milk production, and altered milk composition [36]. Between late pregnancy and early lactation, the MG of control mice showed significant increase in weight, DNA content and levels of lipoprotein lipase (LPL) [37]. The increase in MG weight, DNA content and LPL activity were inhibited in THC-treated mice between late pregnancy and early lactation [37].

No study has evaluated the effect of THC or CBD on alveolar differentiation of MECs. We hypothesized that THC and CBD will impact MEC differentiation and milk protein expression. Furthermore, these effects may be mediated through CB2, as its presence has been previously reported in MECs [38]. Herein, we reported that 10μM THC and 10μM CBD reduced mRNA levels of milk proteins, lactose and lipid synthesizing enzymes, as well as WAP and lipid levels. Furthermore, our results suggested that the impact of THC and CBD on changes in mRNA levels of *CSN2*, *HK2* and *FABP4* may be mediated, in part, via CB2.

## Materials and methods

### Cell culture and differentiation

All work with cells was approved by the McMaster Biosafety committee under Bio Utilization protocol BUP023. As these cells were commercially purchased under a Materials Transfer agreement between McMaster University and the American Type Culture Collection patient consent was not required. HC11 cells were obtained from American Type Culture Collection (CRL-3062) and cultured in RPMI 1640 (Corning, 10-041-CV), supplemented with 10% Fetal Bovine Serum (FBS) (Hyclone, SH30396.03), 1% L-glutamine (Gibco, 25030081) and 1% penicillin-streptomycin (Gibco, 15140122), which formed the base RPMI media. Cells were routinely incubated at 5% CO_2_ and 37°C. For experiments, HC11 cells were seeded at 100,000 cells/cm^2^ in base RPMI media for 24 hours, then supplemented with 10ng/mL epidermal growth factor (EGF) (Thermo Scientific, PMG8041) and 5μg/mL insulin (INS) (Sigma-Aldrich, I0516), for 3 days. The cells were then differentiated with base RPMI media, supplemented with 5μg/mL PRL (PepProTech, 315–16), 1μM dexamethasone (DEX) (Sigma-Aldrich, D4902) and 5μg/mL INS over 4 days. Media was changed every 48 hours, supplemented with appropriate factors at each stage.

Treatment with THC (Sigma-Aldrich, T4764) or CBD (Sigma-Aldrich, C6395) was carried out by supplementing HC11 cells at differentiation with the appropriate concentration of each drug or 0.1% methanol (MeOH) as the vehicle control. For experiments that involved the administration of CB2 agonist, JWH133 (Cayman Chemical, 10005428) or CB2 antagonist, AM630 (Sigma-Aldrich, SML0327), HC11 cells were pre-treated with the agonist/antagonist at differentiation for half an hour followed by treatment with appropriate differentiation factors and drugs in the RPMI base media. Both AM630 and JWH133 were solubilized in dimethyl sulfoxide (DMSO). Therefore, cells were separately treated with 0.1% DMSO at differentiation in the RPMI base media as vehicle control. All experiments were conducted with four to six distinct replicates, which were performed at a different point in time.

### Cytotoxicity and cell proliferation assays

Changes in cellular viability were investigated 4 days post-differentiation, using the CyQUANT lactate dehydrogenase (LDH) Cytotoxicity Assay (LDH assay) (Thermo Fisher, C20300). Furthermore, changes in cellular proliferation were investigated 4 days post-differentiation, using the CellTiter 96 AQueous Non-Radioactive Cell Proliferation Assay (MTS assay) (Promega, G5421). Both assays were conducted according to the manufacturer’s instructions.

### Oil Red O assay

Lipid levels in differentiated HC11 cells were visualized and quantified using an Oil Red O (ORO) assay, a method that has been well characterized for staining and quantifying lipids in fat cells [39]. HC11 cells were washed with Dulbecco’s Phosphate-Buffered Saline (DPBS) (Corning, 20–030) and then fixed with 4% paraformaldehyde (Sigma-Aldrich, P6148-500g) for 45 minutes. Once fixed, the cells were incubated in 60% isopropanol (Sigma-Aldrich, 67-63-0) for 5 minutes and stained with the ORO solution (Sigma-Aldrich, O1391) for 20 minutes. The dye was extracted using 100% butanol (Sigma-Aldrich, B7906) and quantified using a spectrophotometer (Multiskan Spectrum, Thermo Electron Corporation) at 510 nm.

### RNA harvest, cDNA preparation, and RT-qPCR

Harvested cells were resuspended in Trizol (Thermo Fisher Scientific, 15596026), which was extracted with chloroform and eluted through spin columns using the Direct-zol RNA MiniPrep Kit (Cedarlane Labs, R2050). Complementary DNA (cDNA) was prepared using 1μg of total RNA, using the High-capacity cDNA Reverse Transcription Kit (Applied Biosystems, 4368813). Real time quantitative polymerase chain reaction (RT-qPCR) was performed at the following conditions: polymerase activation (95°C for 10 minutes), 39 cycles of denaturing (95°C for 10 seconds), denaturing (60°C for 10 seconds), elongation (72°C for 15 seconds) (Bio-Rad C1000 Touch Thermal Cycler, CFX384 Real-time System). Relative gene expression changes were calculated using the ΔΔCT method, referenced to Ribosomal Protein L0 *(RPL0)* and Ribosomal Protein L8 *(RPL8)* as housekeeping genes. Primer sequences of all the genes investigated in this study, including the housekeeping genes, have been outlined in Table 1.

**Table 1:** Primer sequences of all the genes investigated in this study.

Primer Name	Forward Sequence	Reverse Sequence
***B4GALT1***	TCCCTTTACTCTTGGCAGATTAG	TCTTGTCTCTTGAATGCCGGA
***CNR2***	TGATTGGTGTCAGCTCTCAGT	TTGGTCACTTCTGTCTCCCG
***CSN2***	CCTTCATGGCTGTCAAGTCCT	GTTGTGAGCTGAAGACCACG
***FAAH***	GCTGTGCTCTTTACCTACCTG	GAAGCATTCCTTGAGGCTCAC
***FABP4***	ATTTCCTTCAAACTGGGCGTG	CTTTCCATCCCACTTCTGCAC
***FASN***	CACGAGTGAGTGTACGGGAG	GATCGGAGCATCTCTGGTGG
***GLUT1***	TACACCCCAGAACCAATGGC	CCCGTAGCTCAGATCGTCAC
***HK2***	CATCACGCCAGTGACTCTGATA	AGGTACCCTAAGTCTCACTCCT
***LALBA***	AGCTGGGTCATGTGTGGAAT	GTAATGCAGACACCGCTCAC
***LPL***	ATGGCAAGCAACACAACCAG	AGCAGTTCTCCGATGTCCAC
***NAPEPLD***	TTCTTTGCTGGGGATACTGG	GCAAGGTCAAAAGGACCAAA
***PLIN2***	TAAACGTCTGTCTGGACCGAAT	AAGGAAAAACCTCACCTCAAGC
***RPL0***	CCAGCAGGTGTTTGACAACG	TCCAGAAAGCGAGAGTGCAG
***RPL8***	ACGTGAAGACC GTAAGGG	GATGCCTTTAATGTAGCCGTGT
***WAP***	CAGCCCAAGCCTATACAGCA	TTGCCACCCTGGAGATCCTA

*B4GALT1*: β-1,4-galactosyl transferase; *CNR2*: Cannabinoid receptor 2; *CSN2*: β-casein; *FAAH*: Fatty acid amide hydrolase; *FABP4*: Fatty acid binding protein 4; *FASN*: Fatty acid synthase; *GLUT1*: Glucose transporter 1; *HK2*: Hexokinase 2; *LALBA*: α-lactalbumin; *LPL*: Lipoprotein lipase; *NAPEPLD*: N-acyl phosphatidylethanolamine-specific phospholipase D; *PLIN2*: Perilipin 2; *RPL0*: Ribosomal protein 0; *RPL8*: Ribosomal protein 8; *WAP*: Whey acidic protein.

### Western blotting

Cells were washed with cold DPBS, scraped off in cold Radioimmunoprecipitation assay (RIPA) buffer, and sonicated for 10–15 seconds. Protein concentrations were measured by using the Pierce BCA Protein Assay (Thermo Fisher Scientific, 23227) [40]. 15μg of protein was separated by SDS-PAGE on 10-well, 12% Mini-Protean TGX Stain-free protein gels (Bio-Rad, 4568043), at constant current (0.03 Amps). 10μL of Precision Plus Protein^TM^ All Blue Prestained Protein Standard (Bio-Rad, 1610373) was loaded onto to the gel. Gels were transferred to a Trans-blot turbo Mini 0.2μm polyvinylidene difluoride (PVDF) membrane (Bio-Rad, 1704156), using the transblot apparatus (Bio-Rad), at the following conditions: 21 volts, 1.0 amps, 7 minutes. PVDF membranes were blocked with 5% bovine serum albumin (BSA) (Sigma-Aldrich, A-6003) for 2 hours at room temperature on a shaker, followed by incubation in primary antibody dissolved in 5% BSA, overnight at 4°C with gentle rocking. Following the overnight incubation in the primary antibody, blots were incubated in the appropriate secondary antibody, dissolved in 1.5% BSA, for 2 hours at room temperature. For protein detection, the PVDF membranes were incubated in Clarity Max Western Enhanced Chemiluminescence (ECL) Substrate (Bio-Rad, 1705062) for 3 minutes, and imaged on the ChemiDoc^TM^ Imaging System (Bio-Rad). The full list of concentrations and manufacturers of the primary and secondary antibodies have been outlined in Table 2.

**Table 2:** List of primary and secondary antibodies used in this study.

Antibody Name	Company Name	Catalog Number	Working Dilution
Anti-β-casein mAb	Santa Cruz Biotechnology	sc-166684	1:1000
Anti-WAP mAb	Santa Cruz Biotechnology	sc-374648	1:200
Anti-CB2 pAb	Cayman Chemical Company	101550	1:200
β-Actin Rabbit mAb	Cell Signaling Technology	4970S	1:10,000
Goat Anti-Mouse IgG	Miltenyi Biotec, Inc.	130-048-401	1:5000
Donkey Anti-rabbit IgG	Cytiva	NA9340-1ML	1:5000

The list includes name of the antibody and its manufacturer, catalogue number and concentrations used in this study. CB2: Cannabinoid receptor 2; WAP: Whey acidic protein; mAb: Monoclonal antibody; pAb: Polyclonal antibody.

### Statistical analyses

Statistical analyses were performed using GraphPad Prism software (Prism 9 for macOS, Version 9.1.2 [225]). Data points were expressed as mean ± SEM, and analysed using either a t-test (for <3 test groups), or one-way analysis of variance (ANOVA) (for >3 test groups) with Tukey’s post-test. Results represent data collected from experiments conducted 4 to 6 times. Data points with *p* ≤0.05 will be considered significant and statistical changes were denoted by differing letters on data figures. Different letters (a, b, etc.) indicate a statistically significant difference between two data points. A mix of letters indicate a statistically significant difference between two data points that have distinct set of letters (i.e., ab and cd are statistically different). Meanwhile a mix of letters that have at least one same letter in the combination indicate no significant difference between data points (i.e., ab and b).

## Results

### THC and CBD alter markers of HC11 cell differentiation

**Fig 1:**
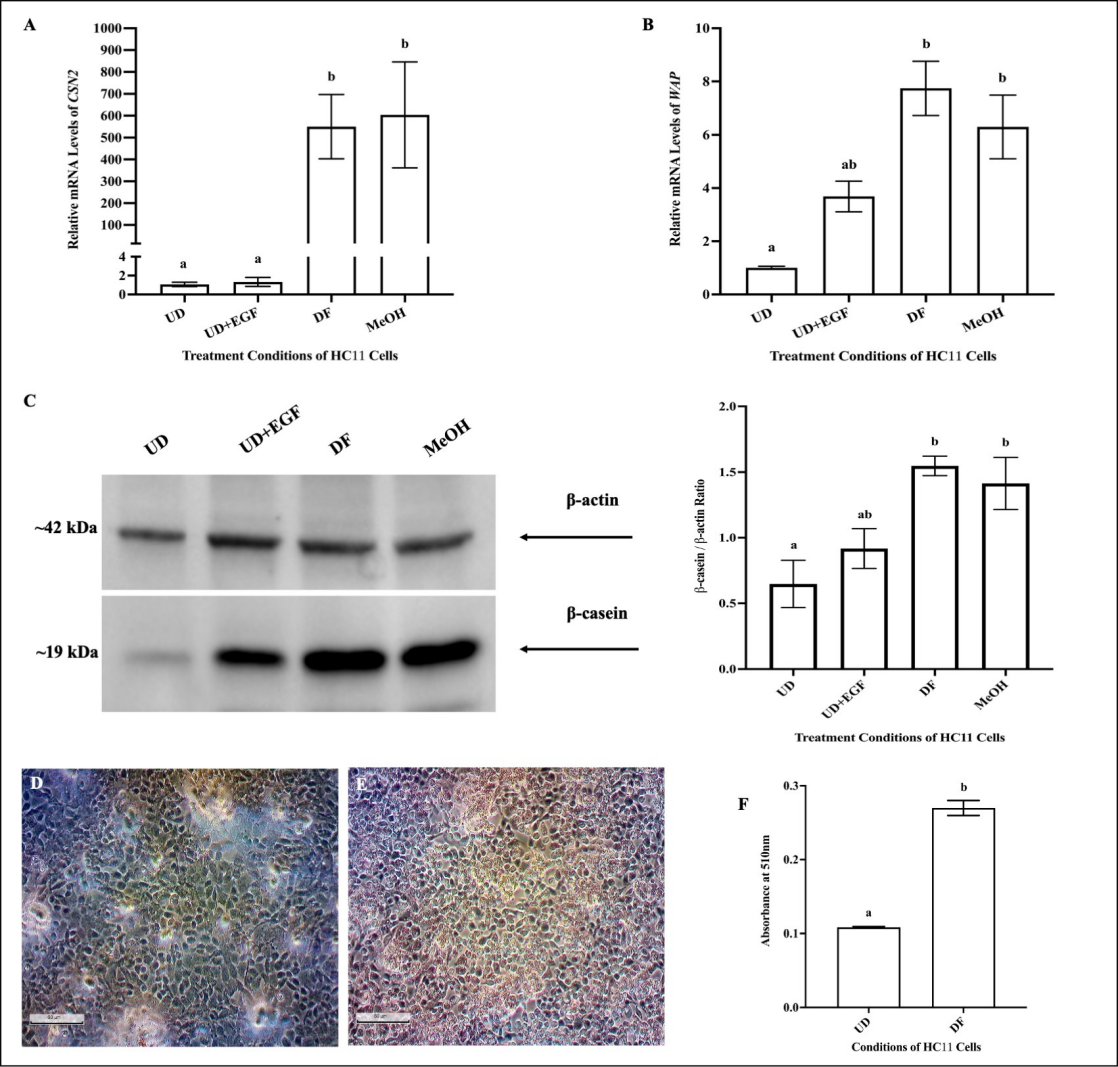
Differentiated HC11 cells displayed increased milk proteins and lipid levels. HC11 cells were seeded in base media for 24 hours (UD). HC11 cells were seeded and supplemented with EGF and INS for 3 days (UD+EGF). Finally, HC11 cells were differentiated in base media containing PRL, INS and DEX, and allowed to proceed for 4 days in the absence (DF) or presence of the vehicle (MeOH). Cells from all conditions were processed for total RNA or protein isolation, as described in methods. The relative (compared to *RPL0 and RPL8*) mRNA levels of *CSN2* (A) and *WAP* (B) were determined. Gene expression analysis was repeated with 6 distinct replicates. Protein expression of β-casein was assessed, as described in methods, using 15μg of total protein for each sample. (C) A representative blot of β-casein expression is shown along with a bar graph quantifying its expression, normalized to β-actin, for undifferentiated (UD), EGF supplemented (UD+EGF), differentiated (DF) and differentiated in the presence of vehicle (MeOH), based on 4 replicates. Lipid levels were assessed in undifferentiated cells (UD) and differentiated cells (DF), by conducting an Oil Red O (ORO) assay, as described in methods. Representative images of ORO-stained undifferentiated HC11 cells (UD) and differentiated cells (DF), taken with a phase-contrast microscope (20x magnification). (F) Lipid levels were quantified in cells, as described in the methods, from 6 distinct replicates of undifferentiated HC11 cells (UD) and differentiated cells (DF). Results were plotted as mean ± SEM and compared using either a student’s t-test (for groups ≤2), or one-way ANOVA (for groups ≥ 3). Statistically significant changes were represented by distinct letters on bar graphs.

Prior to treating cells with THC or CBD, we characterized HC11 differentiation by monitoring changes in gene and protein expression changes of key markers of differentiation, as well as lipid levels. Relative to undifferentiated HC11 cells, differentiated cells displayed significantly higher mRNA levels of *CSN2* and *WAP* (Fig 1A and 1B), and β-casein expression (Fig 1C). HC11 cells differentiated in the presence 0.1% MeOH (vehicle control to THC and CBD) did not display any differences in mRNA levels of milk proteins and β-casein expression, relative to HC11 cells differentiated without MeOH (Fig 1A–1C). EGF and INS, alone, did not result in HC11 cell differentiation, and cells treated with these compounds displayed similar gene and protein levels of milk proteins as undifferentiated cells (Fig 1A–1C). Furthermore, relative to undifferentiated HC11 cells (UD) (Fig 1D), differentiated cells (DF) (Fig 1E) displayed significantly higher lipid accumulation (p-value <0.0001) (Fig 1F).

**Fig 2:**
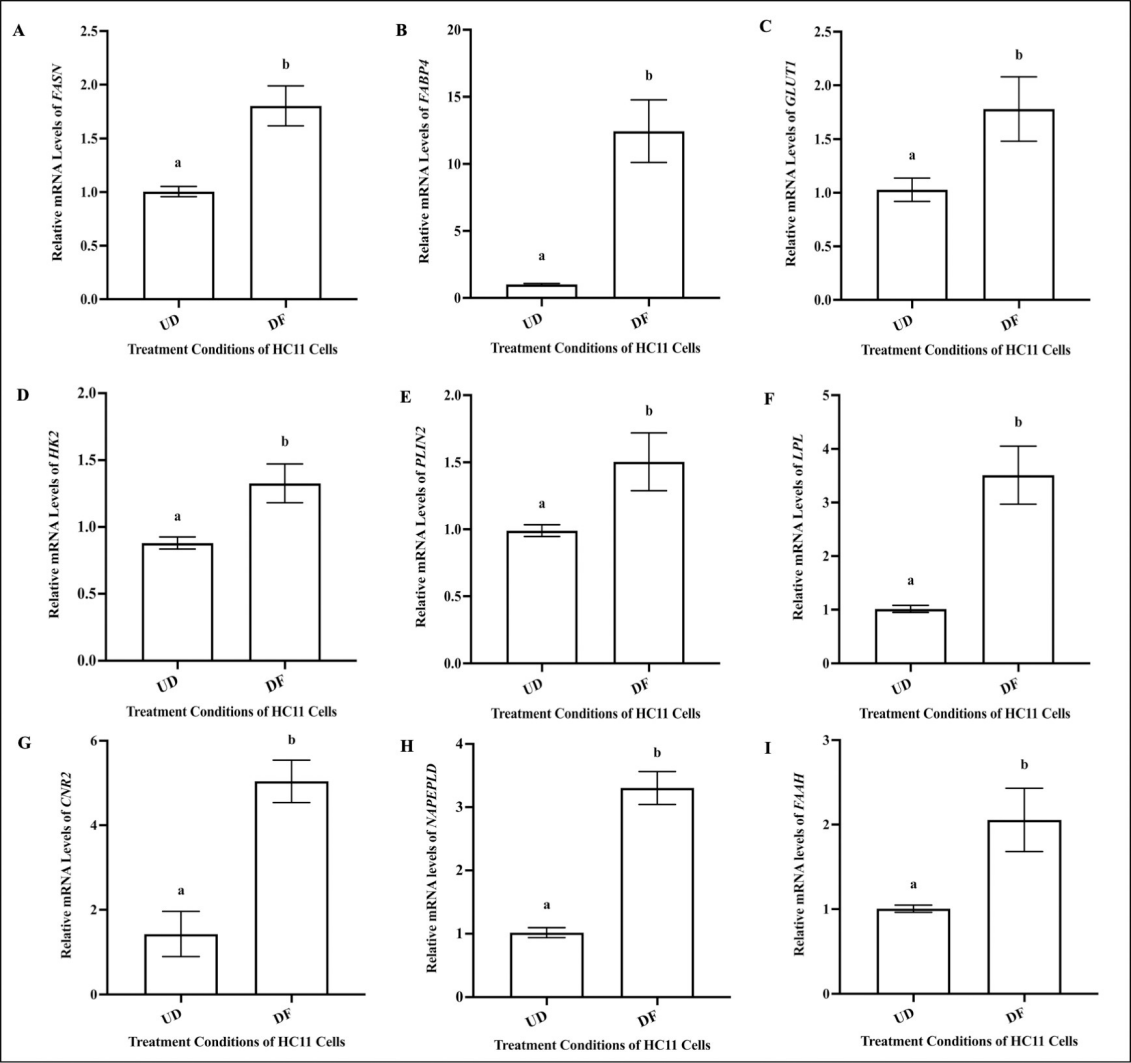
Differentiation increases the expression of mRNA for markers of lipid synthesis and alters mRNA levels of key ECS markers in HC11 cells. HC11 cells were seeded in base media for 24 hours (UD) and collected for RNA isolation. HC11 cells were seeded in base media for 24 hours, supplemented with EGF and INS for 3 days, and differentiated in base media containing PRL, INS and DEX 4 days (DF). Undifferentiated and differentiated cells were processed for total RNA, as described in methods. The relative mRNA levels of *FASN* (A), *FABP4* (B), *GLUT1* (C), *HK2* (D), *PLIN2* (E), *LPL* (F), *CNR2* (G), *NAPE-PLD* (H*)* and *FAAH* (I) were determined. Gene expression analysis was repeated with 6 distinct replicates. Results were represented as mean ± SEM and compared using a t-test. Statistically significant changes were represented by distinct letters on the bar graphs.

Relative to undifferentiated HC11 cells (UD), differentiated cells (DF) demonstrated increased mRNA levels of genes that encode proteins and enzymes responsible for fat synthesis: fatty acid synthase *(FASN)* [41], fat transport: fatty acid binding protein 4 *(FABP4)*, coating lipids: perilipin 2 *(PLIN2)* [42], triglyceride lysis: lipoprotein lipase *(LPL)* [37], and markers that aid in supplying glucose for lactose and lipid synthesis: glucose transporter 1 *(GLUT1)* and hexokinase 2 *(HK2)* [43] (Fig 2A–2F). As for changes in expression of genes that encode major ECS markers, differentiated cells (DF) displayed increased levels of *CNR2* (the gene that encodes CB2) and *N*-acyl phosphatidylethanolamine-phospholipase D *(NAPEPLD)*, no change in fatty acid amide hydrolase (*FAAH)* levels, relative to undifferentiated cells (UD) (Fig 2G–2I).

### High concentrations of THC and CBD reduce HC11 cell proliferation and viability

**Fig 3:**
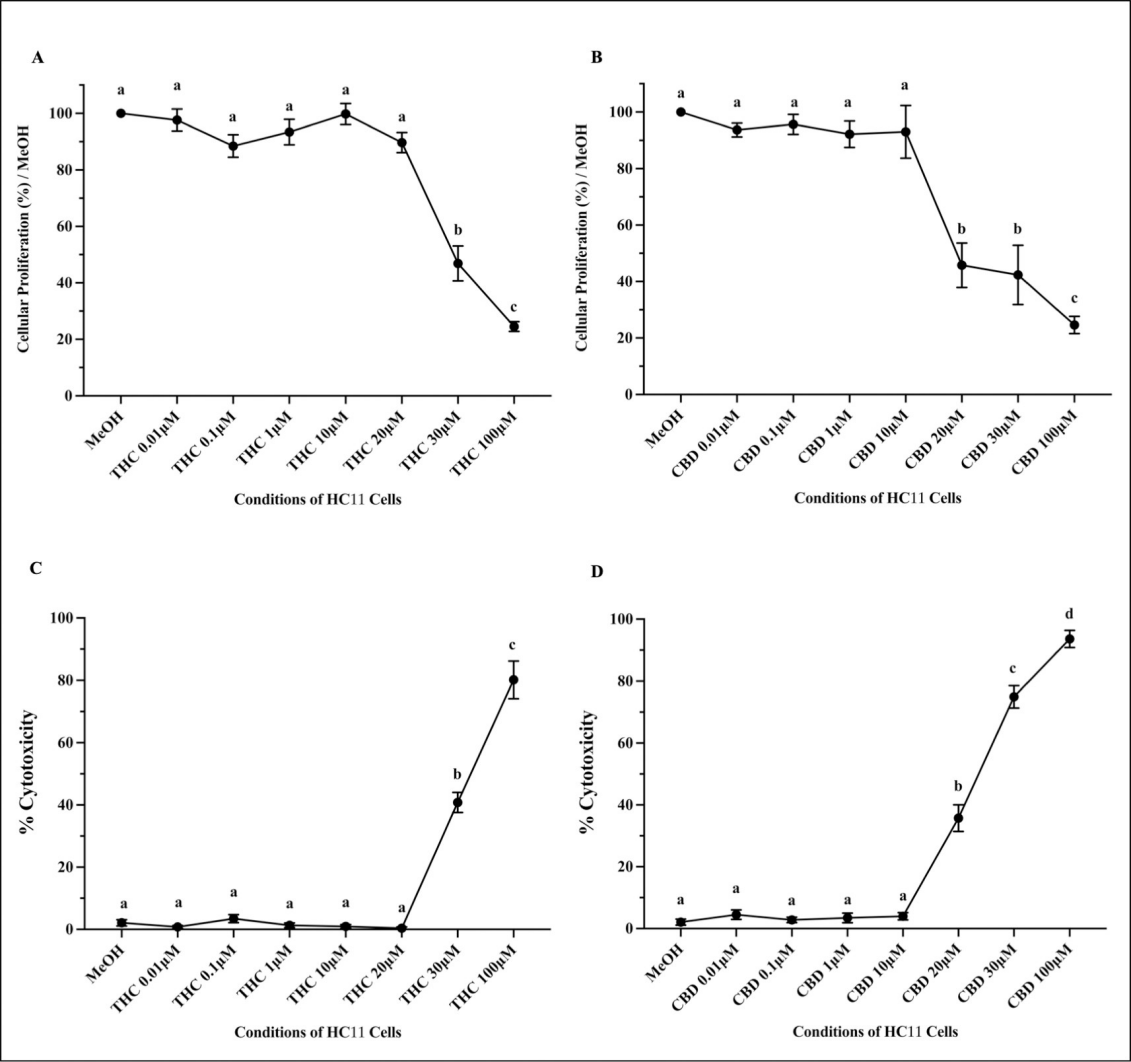
High concentrations of THC and CBD reduced proliferation and viability of HC11 cells. HC11 cells were seeded in base media for 24 hours, supplemented with EGF and INS for 3 days, and differentiated in base media containing PRL, INS and DEX, as described in methods, and treated with vehicle control (MeOH), or 0.01, 0.1, 1, 10, 20, 30, 100μM THC (A and C) or CBD (B and D) for 4 days. Changes in cellular proliferation and toxicity were evaluated using a MTS assay and LDH assay, respectively, as described in methods. (A-B) Changes in absorbances were recorded following a MTS assay and results were normalized to vehicle control (MeOH) as a percentage. (C-D) The relative cytotoxicity of THC and CBD was assessed using a LDH assay and percent cytotoxicity (compared to the vehicle control) was plotted. All experiments were repeated with 6 distinct replicates. Results were represented as mean ± SEM and compared using one-way ANOVA. Statistically significant changes were indicated by distinct letters.

The impact of various concentrations of THC and CBD (0.1, 0.1, 1, 10, 20, 30 and 100 μM), independently, was investigated on cytotoxicity and proliferation of HC11 cells during differentiation (Fig 3). Relative to vehicle control (MeOH), 30μM and 100μM THC (Fig 3A), as well as well as 20μM, 30μM and 100μM CBD (Fig 3B), significantly reduced HC11 cell proliferation. Following the MTS assay, absorbance values were recorded, normalized to the vehicle control, and represented as a percentage in the bar graphs (Fig 3A and 3B). In addition, relative to vehicle control (MeOH), 30μM and 100μM THC (Fig 3C), as well as well as 20μM, 30μM and 100μM CBD (Fig 3D), significantly induced cellular toxicity in HC11 cells. Cellular toxicity was measured using a LDH assay and calculated according to the manufacturer’s directions. Based on these results, all following experiments were conducted using 10μM THC and 10μM CBD.

### THC and CBD exhibit a differential effect on β-casein protein levels

**Fig 4:**
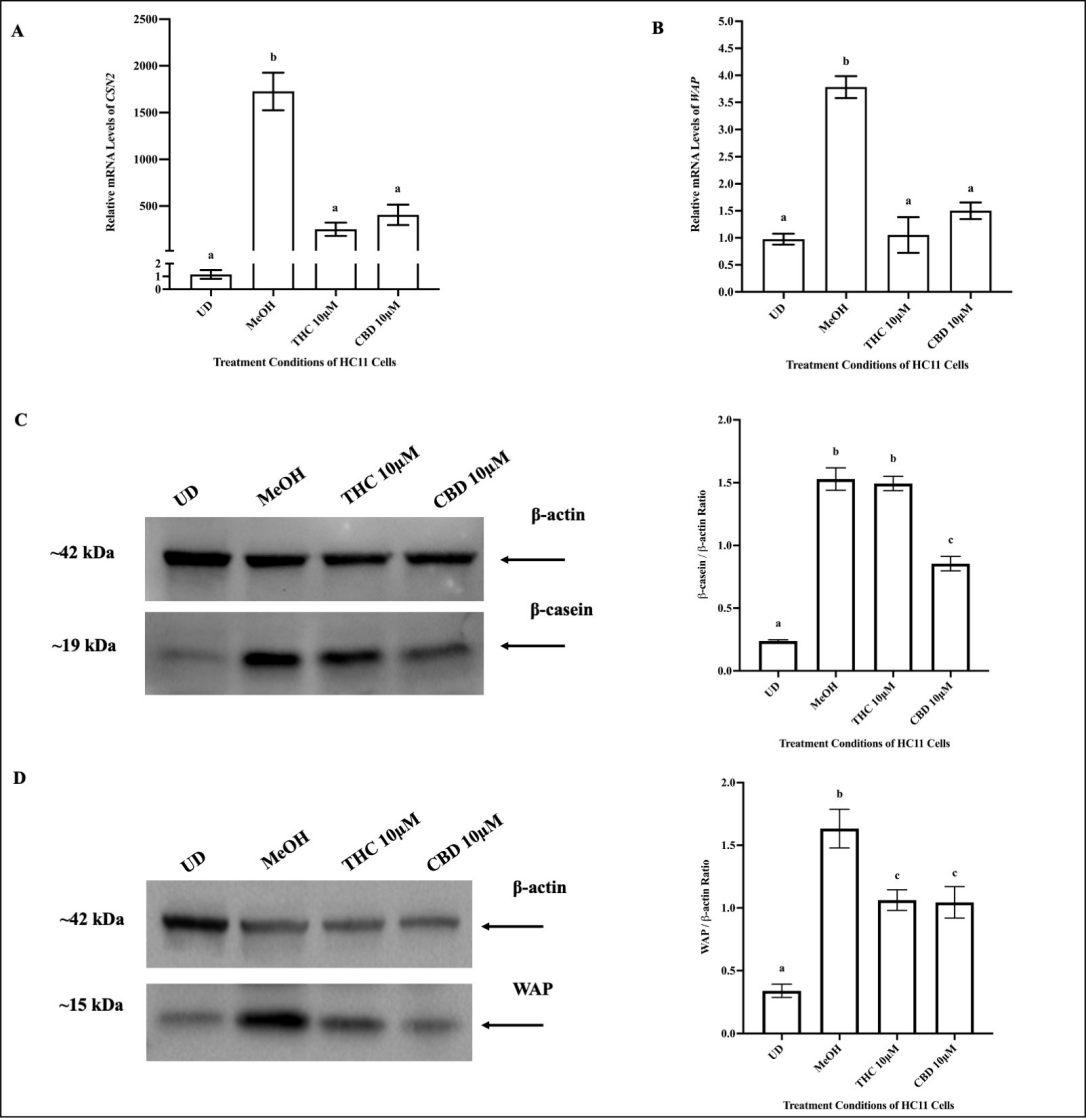
10μM THC and 10μM CBD reduced levels of milk proteins in HC11 cells. HC11 cells were seeded in base media for 24 hours (UD) and processed for total RNA or protein, as described in the methods. HC11 cells were differentiated in base media containing PRL, INS and DEX, as described in methods, and treated with vehicle control (MeOH), 10μM THC or 10μM CBD for 4 days, and processed for total RNA or protein. The relative mRNA levels of *CSN2* (A) and *WAP* (B) were determined. Gene expression analysis was repeated with 6 distinct replicates. Milk protein expression was assessed, as described in the methods, using 15μg of total protein for each sample. Representative blots of β-casein (C) and WAP (B) are shown along with a bar graph quantifying the protein expression, normalized to β-actin, for undifferentiated cells (UD), vehicle control (MeOH), 10μM THC and 10μM CBD treated HC11 cells, based on 4 replicates. Results were plotted as mean ± SEM and compared using one-way ANOVA. Statistically significant changes were represented by distinct letters.

Relative to vehicle control, 10μM THC and 10μM CBD significantly downregulated the mRNA levels of *CSN2* and *WAP* (Fig 4A and 4B). The mRNA levels of *CSN2* and *WAP* in cells treated with 10μM THC and CBD were similar to levels seen in undifferentiated cells (UD) (Fig 4A and 4B). To validate the changes observed in mRNA’s encoding milk proteins upon THC and CBD treatment, protein expression of β-casein and WAP was quantified in HC11 cells and normalized to β-actin. Relative to vehicle control, THC-treated cells did not display any changes in β-casein expression (Fig 4C) but displayed significantly lower WAP expression (Fig 4D). HC11 cells treated with CBD displayed decreased expression of both β-casein (Fig 4C) and WAP (Fig 4D), relative to vehicle control cells. Compared to THC, CBD-treated cells demonstrated a significant decrease in β-casein expression (Fig 4C), but no change in WAP expression (Fig 4D).

### THC and CBD alter the gene expression of lactose and lipid synthesizing enzymes, and reduce lipid levels in HC11 cells

**Fig 5:**
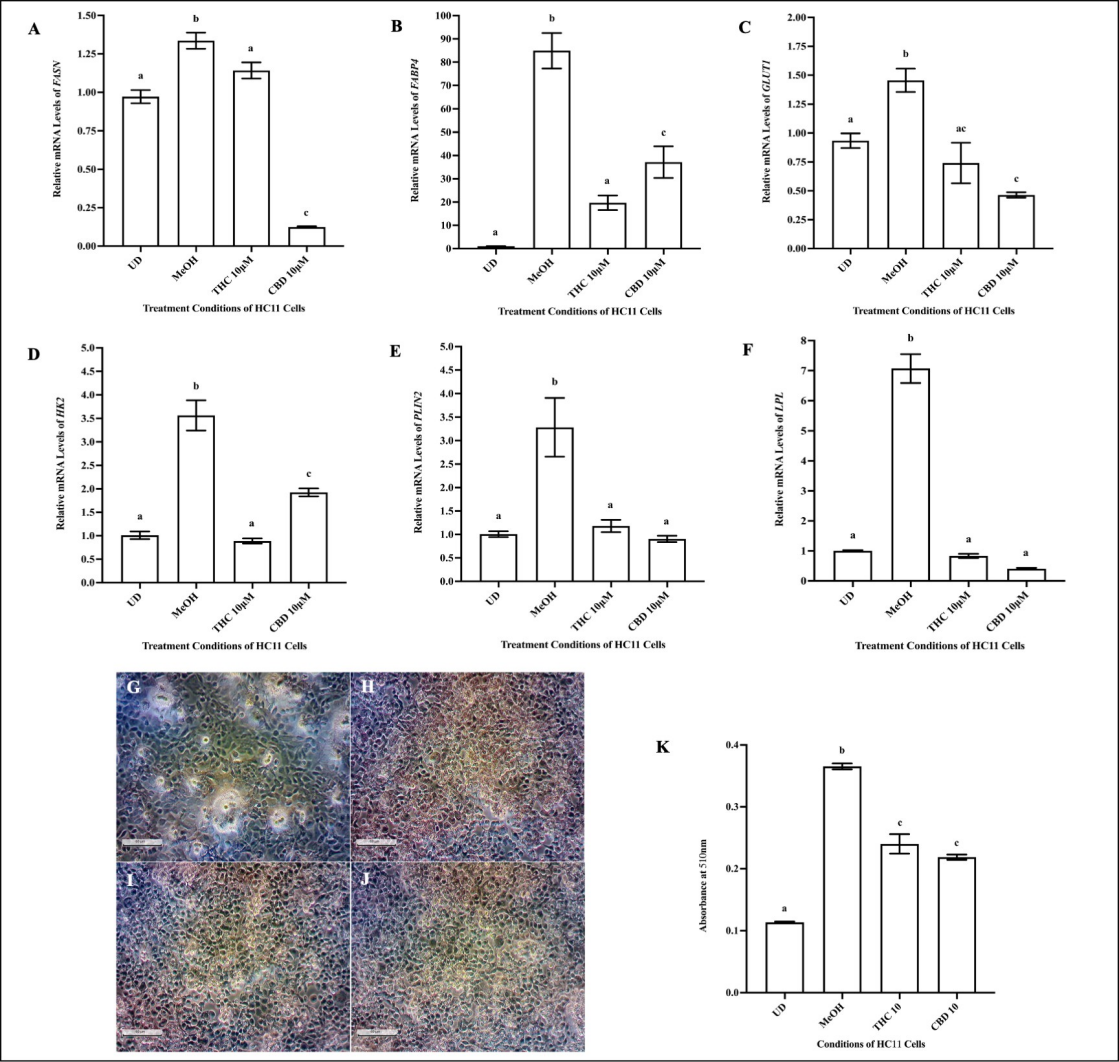
10μM THC and 10μM CBD reduced lipid levels and mRNA levels of associated markers in HC11 cells. HC11 cells were seeded in base media for 24 hours (UD) and processed for total RNA isolation, as described in the methods. HC11 cells were differentiated in base media containing PRL, INS and DEX, as described in methods, and treated with vehicle control (MeOH), 10μM THC or 10μM CBD for 4 days, and processed for total RNA isolation. The relative mRNA levels of *FASN* (A), *FABP4* (B), *GLUT1* (C), *HK2* (D), *PLIN2* (E) and *LPL* (F) were determined. Gene expression analysis was repeated with 6 distinct replicates. Lipid levels in cells were quantified by conducting an Oil Red O (ORO) assay, as described in methods. Representative images of ORO-stained undifferentiated (UD) cells (G), and cells differentiated with vehicle control (MeOH) (H), 10μM THC (I), and 10μM CBD (J), were taken using a phase-contrast microscope (20x magnification). (K) Lipid levels were quantified in undifferentiated (UD) cells, and cells treated with vehicle control (MeOH), 10μM THC or 10μM CBD, from 6 distinct replicates. Results were plotted as mean ± SEM and compared using one-way ANOVA. Statistically significant changes were represented by distinct letters on bar graphs.

Relative to vehicle control, 10μM THC and 10μM CBD significantly decreased mRNA levels of *FASN*, *FABP4*, *GLUT1*, *HK2*, *PLIN2* and *LPL* (Fig 5A–5F). THC decreased the mRNA levels of *FABP4* and *HK2* to a greater extent than CBD (Fig 5B and 5D). In contrast, CBD downregulated *FASN* gene expression more profoundly than THC (Fig 5A). Relative to undifferentiated HC11 cells (Fig 5G) and differentiated vehicle control cells (Fig 5H), the cells treated with 10μM THC (Fig 5I) and 10μM CBD (Fig 5J) demonstrated significantly lower lipid levels after 4 days of differentiation (Fig 5K).

### THC and CBD treatment results in altered expression of CB2 and endocannabinoid metabolizing mRNA markers in HC11 cells

**Fig 6:**
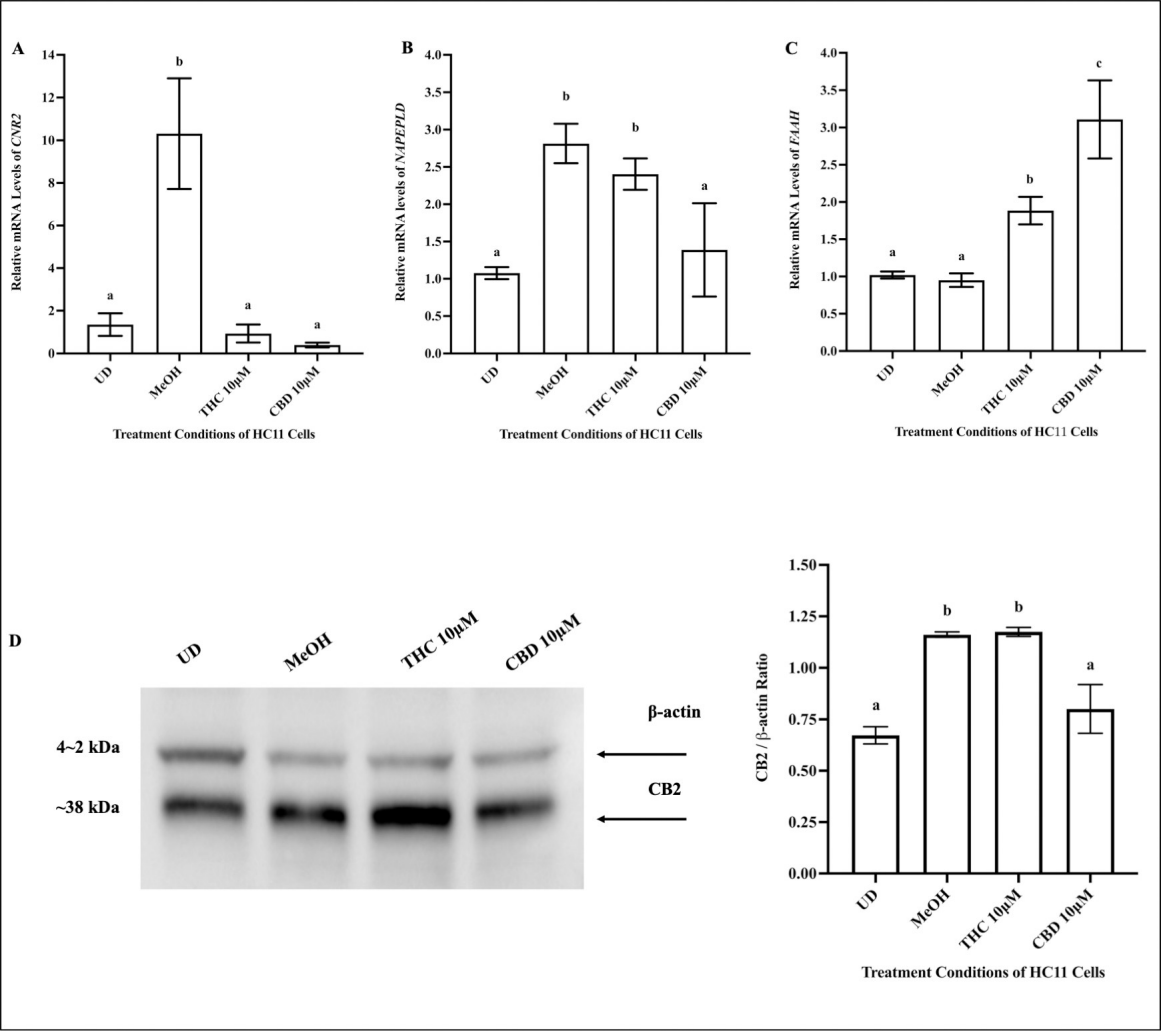
10μM THC and 10μM CBD altered mRNA levels of ECS markers in HC11 cells. HC11 cells were seeded in base media for 24 hours (UD) and processed for total RNA or protein isolation, as described in the methods. HC11 cells were differentiated in base media containing PRL, INS and DEX, and treated with vehicle control (MeOH), 10μM THC or 10μM CBD for 4 days. Treated cells were processed for total RNA or protein isolation, as described in methods. The relative mRNA levels of *CNR2* (A), *NAPEPLD* (B) and *FAAH* (C) were determined. Gene expression analysis was repeated with 6 distinct replicates. Milk protein expression was assessed, as described in methods, using 15μg of total protein for each sample. (D) A representative blot of CB2 expression is shown along with a bar graph quantifying its expression, normalized to β-actin, for undifferentiated (UD), and differentiated cells in the presence of vehicle (MeOH), 10μM THC, or 10μM CBD, based on 4 replicates. Results were plotted as mean ± SEM and compared using one-way ANOVA. Statistically significant changes were represented by distinct letters on bar graphs.

Relative to vehicle control, 10μM THC significantly decreased mRNA levels of *CNR2* (Fig 6A), did not change *NAPEPLD* mRNA levels (Fig 6B), and significantly increased *FAAH* mRNA levels in HC11 cells (Fig 6C) in HC11 cells. In contrast, 10μM CBD significantly downregulated the gene expression of *CNR2* and *NAPEPLD* (Fig 6A and 6B) and upregulated the gene expression of *FAAH* (Fig 6C), compared to vehicle control. Changes in the mRNA levels of *CNR2* were further validated by conducting protein expression analysis. Relative to undifferentiated cells (UD), cells differentiated in the presence of the vehicle control (MeOH) demonstrated significant upregulation in CB2 expression (Fig 6D). Compared to vehicle control (MeOH), THC treatment did not alter CB2 expression, while CBD significantly downregulated the levels of CB2 in HC11 cells (Fig 6D).

### CB2 receptor contributes to effects of THC and CBD on milk protein and lipid synthesis

**Fig 7:**
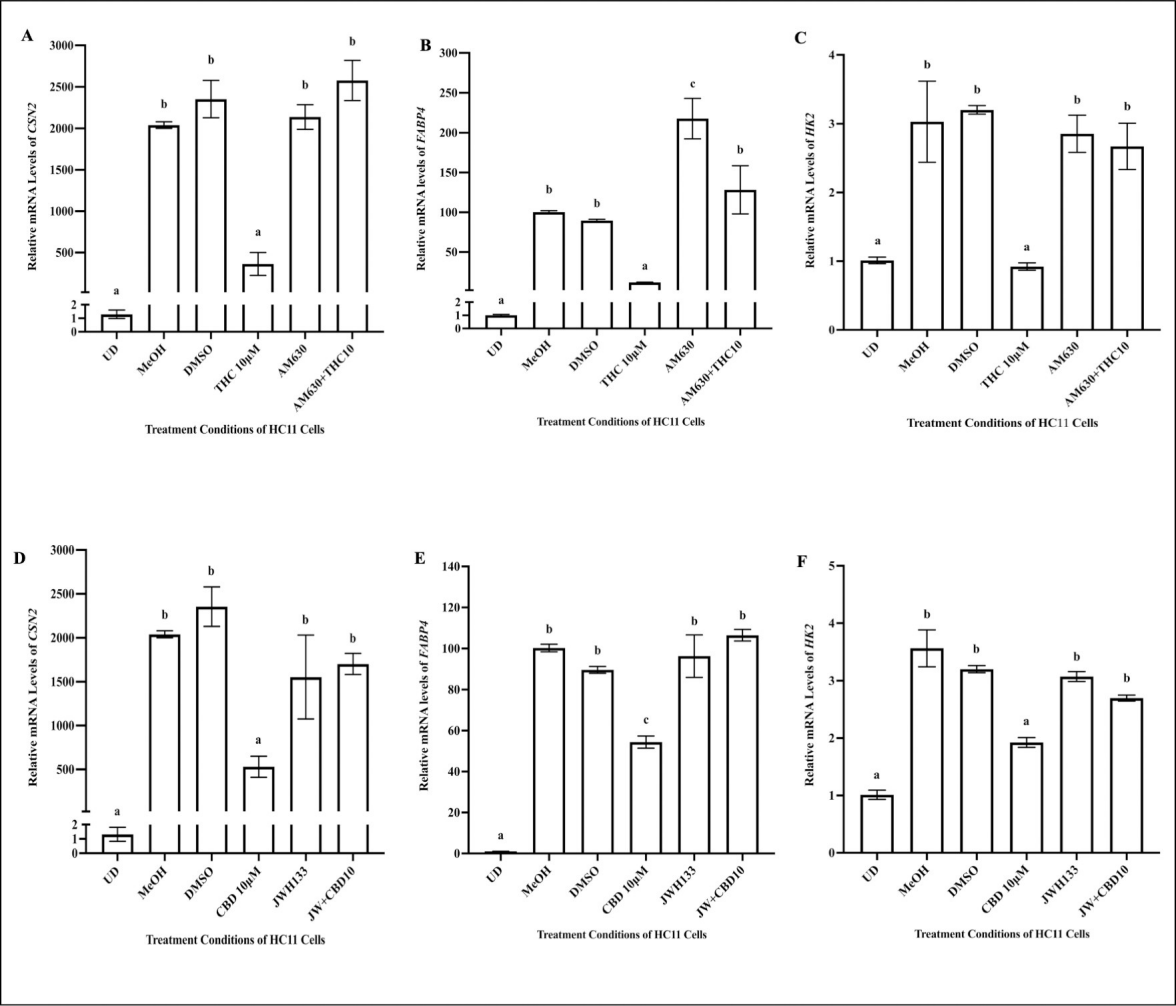
AM630 and JWH133 rescued the effect of THC and CBD, respectively, in HC11 cells. HC11 cells were seeded in base media for 24 hours (UD) and processed for total RNA isolation, as described in the methods. HC11 cells were differentiated in base media containing PRL, INS and DEX, as described in methods, and treated with vehicle controls (MeOH, DMSO), 10μM THC, 1μM AM630, and 1μM AM630 + 10μM THC for 4 days, during cellular differentiation and processed for total RNA isolation. The relative mRNA levels of *CSN2* (A), *HK2* (B) and *FABP4* (C) were determined. Next, the HC11 cells were differentiated in base media containing PRL, INS and DEX, and treated with vehicle controls (MeOH, DMSO), 10μM CBD, 10μM JWH133, and 10μM JWH133 + 10μM CBD for 4 days, and processed for total RNA isolation. The relative mRNA levels of *CSN2* (D), *HK*2 (E) and *FABP4* (F) were quantified. Gene expression analysis was repeated with 6 distinct replicates, as described in methods. Results were represented as mean ± SEM, and compared using one-way ANOVA. Statistically significant changes were indicated by distinct letters on the bar graphs.

Next, we assessed whether co-treatment of HC11 cells with THC AM630, a synthetic antagonist of CB2 [44], would reverse the impact of THC on mRNA levels of *CSN2*, *HK2* and *FABP4* in HC11 cells (Fig 7A–7C). In addition, we investigated whether co-treatment of HC11 cells with CBD and JWH133, a potent selective CB2 agonist [45], will reverse the impact of CBD on mRNA levels of *CSN2*, *HK2* and *FABP4* (Fig 7D–7F). As DMSO is the vehicle for AM630 and JWH133, a set of HC11 cells were differentiated with 0.1% DMSO, as an additional vehicle control. Relative to undifferentiated cells (UD), MeOH- and DMSO-treated differentiated controls displayed significantly increased *CSN2*, *HK2* and *FABP4* mRNA levels (Fig 7). While THC reduced the mRNA levels of *CSN2*, *HK2* and *FABP4* relative to differentiated controls (MeOH, DMSO), AM630 co-treated with THC reversed the effects of THC to levels comparable to those seen in vehicle treated cells (MeOH, DMSO) (Fig 7A–7C). Similarly, CBD downregulated the gene expression of *CSN2*, *HK2* and *FABP4*, relative to differentiated control cells (MeOH, DMSO). Co-treatment of HC11 cells with JWH133 and CBD reversed the effects of CBD, on all tested genes, to levels comparable to those seen in vehicle treated cells (MeOH, DMSO) (Fig 7D–7F).

## Discussion

While the concentrations of THC and CBD used in this study are higher than those reported in human plasma [46,47] and maternal hair [48], they remain on the lower end of concentrations used *in-vitro*. However, the average THC content in cannabis has increased from 4% to 12% over the past two decades [49]. In fact, levels as high as 30% of THC have been reported in legal cannabis grown for recreational use [50]. In rats, daily administration of THC resulted in ten times greater concentrations of THC in fat, compared to any other tissues investigated, and the drug persisted in fat for 2 weeks [51]. Furthermore, Rawitch et al demonstrated that while THC was detected at 200 ng/g, 100 ng/g, and 30 ng/g in the liver, lung and brain of mice, respectively, the levels of THC in the gonadal fat organ were detected between 2000 and 3000 ng/g of tissue [52]. As MECs synthesize and store lipid [53], these cells may sequester, and be exposed to, higher concentrations of cannabinoids than those reported in plasma. *In vitro* studies have used concentrations between 10μM and 40μM to explore the impact of THC [54–56] and CBD [29,57] on proliferation and differentiation in various tissue types. In our study, the HC11 cells were treated with 10μM THC and 10μM CBD.

HC11 cells originate from midpregnant BALB/c mouse MG tissue, and upon treatment with PRL, DEX and INS, these cells adopt a secretory phenotype that resembles differentiated MECs found in alveoli of a lactating MG [35,58,59]. 10μM THC and CBD reduced *CSN2* mRNA levels, relative to vehicle control treated HC11 cells. *CSN2* encodes for β-casein, which is an extremely important milk protein for infant health due to its ability to aid in digestion and absorption of macronutrients and micronutrients [60]. β-casein is digested to phosphopeptides, which have antithrombotic, antihypertensive, and opioid activities that contribute to maintaining proper sleep behaviour and decreasing risk of hypertension in infants [61]. Decreased *CSN2* levels in mammary tissue of mice have been associated with morphological changes in the MG during peak lactation [62]. Distorted MG architecture and less dense alveoli have been accompanied with decreased β-casein and WAP levels in the milk of mice [63]. Decreased expression of *CSN2* in MECs has been demonstrated to be consistent with altered differentiation of MECs [64].

WAP is the principal whey protein in rodent milk, its mRNA levels in mice have been reported to increase by 30 to 50-fold over gestation [65], and its presence in MECs is indicative of terminal differentiation [59]. Overexpression of WAP transgene impaired lobulo-alveolar development in the MG of mice [66], and its forced expression in HC11 cells inhibited MEC proliferation [67]. WAP has been reported to regulate short- and long-term food intake by inducing satiety [68], have potent antimicrobial and protease inhibitory activity [69], and is being investigated for its key roles in immunity [70]. In this study, reduction in gene and protein expression of WAP suggests that THC and CBD may negatively impact terminal differentiation of MECs, and potentially result in lower WAP levels in the milk of those exposed to THC or CBD.

The decrease in mRNA levels of markers involved in lipid and glucose, caused by THC and CBD, was accompanied by reduced lipid levels in HC11 cells treated with 10μM THC and 10μM CBD. In milk, lipids are the primary triglycerides that contribute a sizable percentage of calories, essential fatty acids, and bioactive components, which are required for neonatal growth and development [71]. Decreased expression of markers responsible for glucose uptake *(GLUT1)*, fat transport *(FABP4)* and fatty acid synthesis *(FASN)*, accompanied with reduced lipid levels may lead to lower lipid synthesis and secretion by MECs. Altogether, our results demonstrate that 10μM THC and 10μM CBD impact HC11 cell differentiation, determined by reduced gene expression of milk proteins and lipid synthesizing markers, decreased milk protein (WAP) and lipid levels. However, the effect of cannabis or its metabolites on mammary gland remodeling during pregnancy and on breast milk composition needs to be confirmed by *in vivo* work using animal models and clinical studies in women who use cannabis during the entire perinatal period vs just during lactation.

Exogenous cannabinoids, such as THC and CBD, impact the growth and function of cells through various mechanisms, including via cannabinoid receptors (CBs). Previous studies exploring CB distribution in peripheral cells have reported the presence of CB2 in MECs [38,72]. CB2 is a G protein-coupled receptor (GPCR), and its activation or inhibition results in alterations to cellular physiology, including changes in synaptic function, proliferation, and differentiation [73]. Relative to undifferentiated cells, differentiated HC11 cells displayed increased *CNR2* and *NAPEPLD* levels (the gene that encodes the enzyme that synthesizes AEA). AEA is a weak agonist of CB2 and an important intermediate in lipid metabolism [25]. Therefore, upregulation of *CNR2* and *NAPEPLD* levels may be required in alveolar differentiation for AEA uptake in MECs.

Similar to AEA, THC and CBD are stored in fat depots and activate CB receptors, but neither THC nor CBD are vulnerable to degradation by FAAH [25]. THC has been reported to be a partial agonist of CB2 and is known to compete with endogenous ligands, such as AEA [74]. THC treatment of HC11 cells, reduced the expression of *CNR2*, but the protein expression of the CB2 receptor was unaltered during 4 days of cannabinoid treatment. It is possible that CB2 expression changes may be transient or take longer to manifest, which should be investigated in the future. Moreover, our results demonstrated that the impact of THC on HC11 cell differentiation may be mediated via CB2. When co-treated with THC, AM630 (a selective antagonist of CB2 [75]) restored the levels of *CSN2*, *HK2* and *FABP4*, which had decreased upon THC treatment. In contrast to THC, CBD has been reported to be a potent inverse agonist of CB2 [76]. An inverse agonist competes with an agonist for the orthosteric binding site on a receptor [76]. CBD may compete with endogenous ligands of CB2, resulting in reduced activity of the CB2 receptor and reduction in its gene and protein expression. Moreover, the selective CB2 agonist, JWH133 [77], rescued the effects of CBD on *CSN2*, *HK2* and *FABP4* mRNA levels, when co-treated with CBD.

In addition, THC upregulated mRNA levels of *FAAH*, suggesting that THC may be resulting in breakdown of AEA. CBD downregulated *NAPEPLD* levels and upregulated mRNA levels of *FAAH*, which suggests that treatment of HC11 cells with CBD may result in decreased synthesis and increased breakdown of AEA in HC11 cells. Altogether, the evidence suggests that THC and CBD alter canonical ECS signaling within HC11 cells. Furthermore, reversal of the effects of THC and CBD following co-treatment with a CB2 receptor antagonist and agonist, respectively, indicates that THC and CBD may be impacting HC11 cell differentiation, in part through CB2. However more detailed investigation is required to further elucidate these findings.

## Supporting information

S1 Raw images (PDF)
