# Supplementary material for: Effect of Delta-9-tetrahydrocannabinol and cannabidiol on milk proteins and lipid levels in HC11 cells
Source: PLoS One. 2022 Aug 17;17(8):e0272819. doi: 10.1371/journal.pone.0272819 (PMC9384983; doi:10.1371/journal.pone.0272819)
Supplement: S1 Raw images — (PDF) [file pone.0272819.s001.pdf]

A

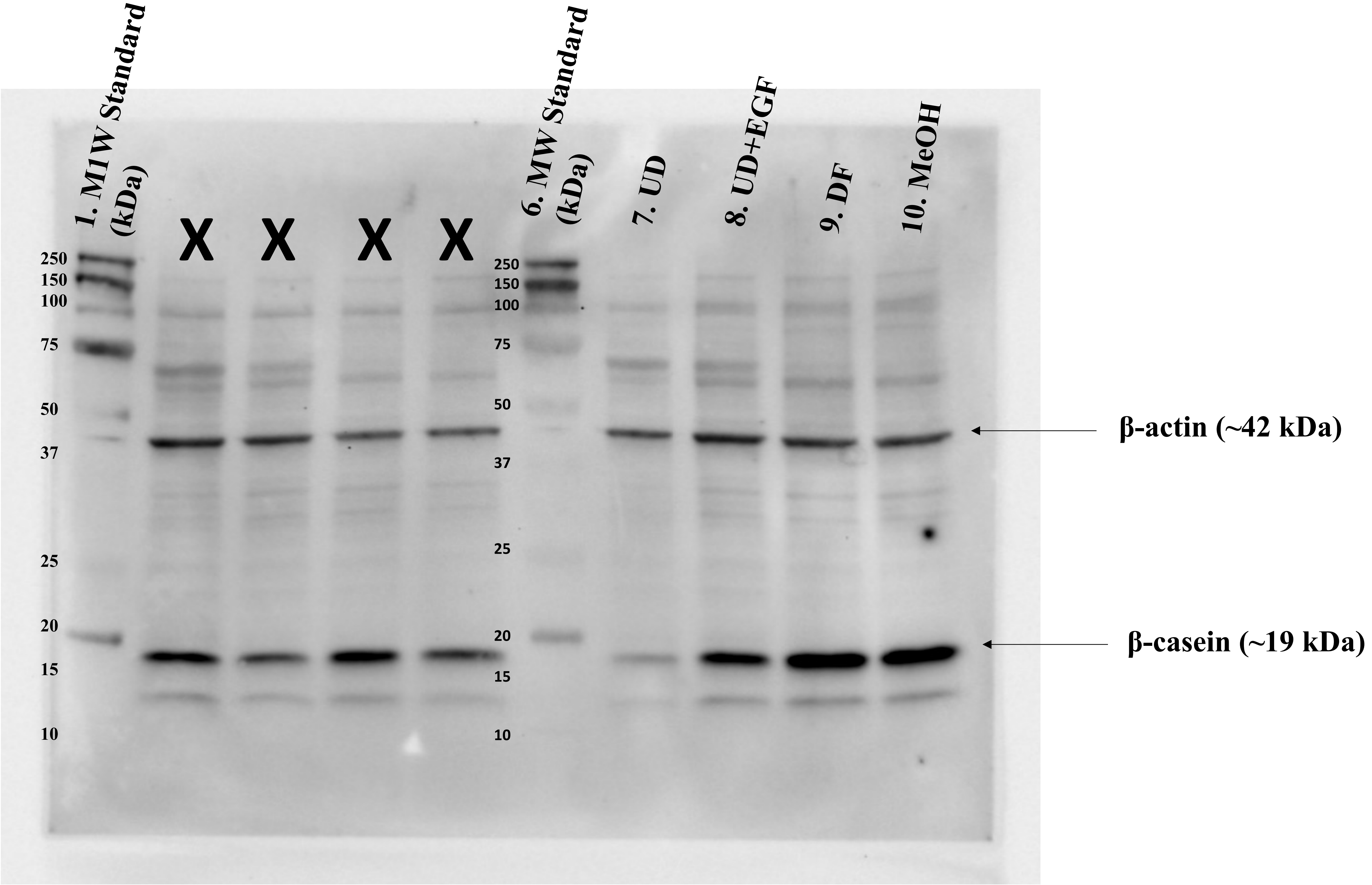

S1 Fig 1. Differentiated HC11 cells displayed increased β-casein levels in HC11 cells, normalized to β-actin. HC11 cells were seeded in base media for 24 hours (UD). HC11 cells were seeded and supplemented with EGF and INS for 3 days (UD+EGF). Finally, HC11 cells were differentiated in base media containing PRL, INS and DEX, and allowed to proceed for 4 days in the absence (DF) or presence of the vehicle (MeOH). Cells from all conditions were processed for total protein isolation, as described in methods. For each sample, 15μg of total protein was separated on 12%, 10-well protein gel. The gel was transferred onto a PVDF membrane, blocked with 5% BSA for 2 hours at room temperature, blocked with 1:1000 anti-β-casein overnight at 4°C with gentle rocking. Next day, the PVDF membrane was incubated in 1:10000 anti-β-actin for 2 hours at room temperature, followed by incubation in 1:5000 of Goat Anti-Mouse IgG for 2 hours at room temperature. The proteins were detected by incubation of the PVDF membranes in ECL for 3 minutes and imaged using the Bio-Rad ChemiDoc™ Imaging System. (A) A representative blot of β-casein is shown for undifferentiated (UD), EGF supplemented (UD+EGF), differentiated (DF) and differentiated cells in the presence of vehicle (MeOH). Figure 1C was generated from lanes 7-10 in this data set. Comparative analysis was performed between UD, UD+EGF, DF and MeOH. Molecular weight (MW) standards have been labeled in lanes 1 and 6. Lanes 2-5 have been marked with an "X" as they were not included in the final figure reported in this study.

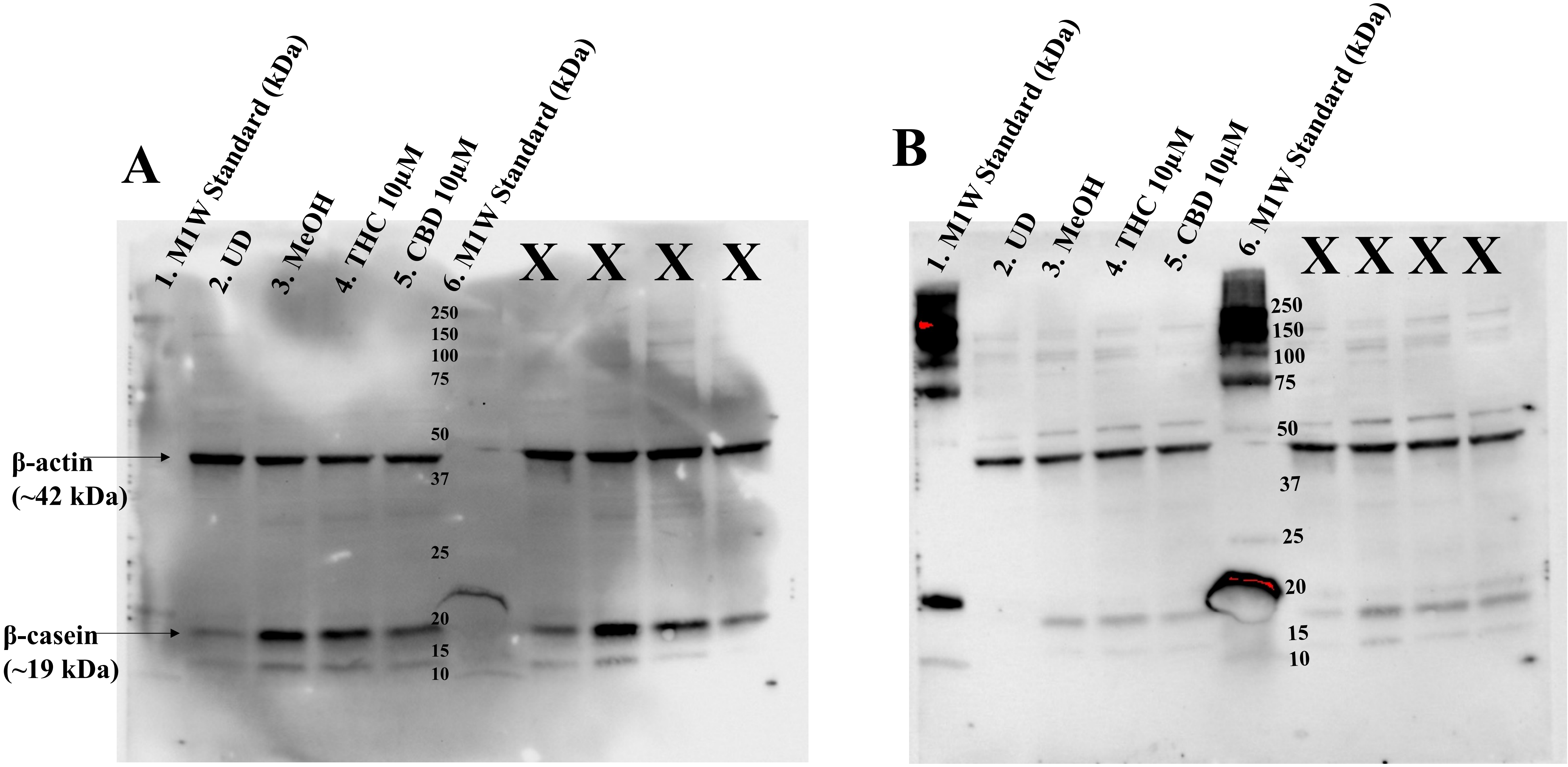

S1 Fig 2. 10μM THC and 10μM CBD reduced β-casein levels in HC11 cells, normalized to β-actin. HC11 cells were seeded in base media for 24 hours (UD), as described in the methods. HC11 cells were seeded in base media for 24 hours, supplemented with EGF and INS for 3 days, and differentiated in base media containing PRL, INS and DEX, as described in methods, and treated with either vehicle control (MeOH), 10μM THC or 10μM CBD for 4 days, as described in methods. Cells from all conditions were processed for total protein isolation, as described in methods. For each sample, 15μg of total protein was separated on 12%, 10-well protein gel. The gel was transferred onto a PVDF membrane, blocked with 5% BSA for 2 hours at room temperature, blocked with 1:1000 anti-β-casein overnight at 4ºC with gentle rocking. Next day, the PVDF membrane was incubated in 1:10000 anti-β-actin for 2 hours at room temperature, followed by incubation in 1:5000 of Goat Anti-Mouse IgG for 2 hours at room temperature. The proteins were detected by incubation of the PVDF membranes in ECL for 3 minutes and imaged using the Bio-Rad ChemiDoc™ Imaging System. (A) Representative blot of β-casein and β-actin expression in undifferentiated HC11 cells (UD), and cells treated with the vehicle control (MeOH), 10μM THC and 10μM CBD. Figure 4C was generated from lanes 2-5 in this data set. (B) Same membrane as (A), imaged at a different conditions, provided for clearer representation of the Molecular Weight (MW) standards. Comparative analysis was performed between UD, MeOH, 10μM THC and 10μM CBD. Molecular weight (MW) standards have been labeled in lanes 1 and 6. Lanes 7-10 have been marked with an "X" as they were not included in the final figure reported in this study.

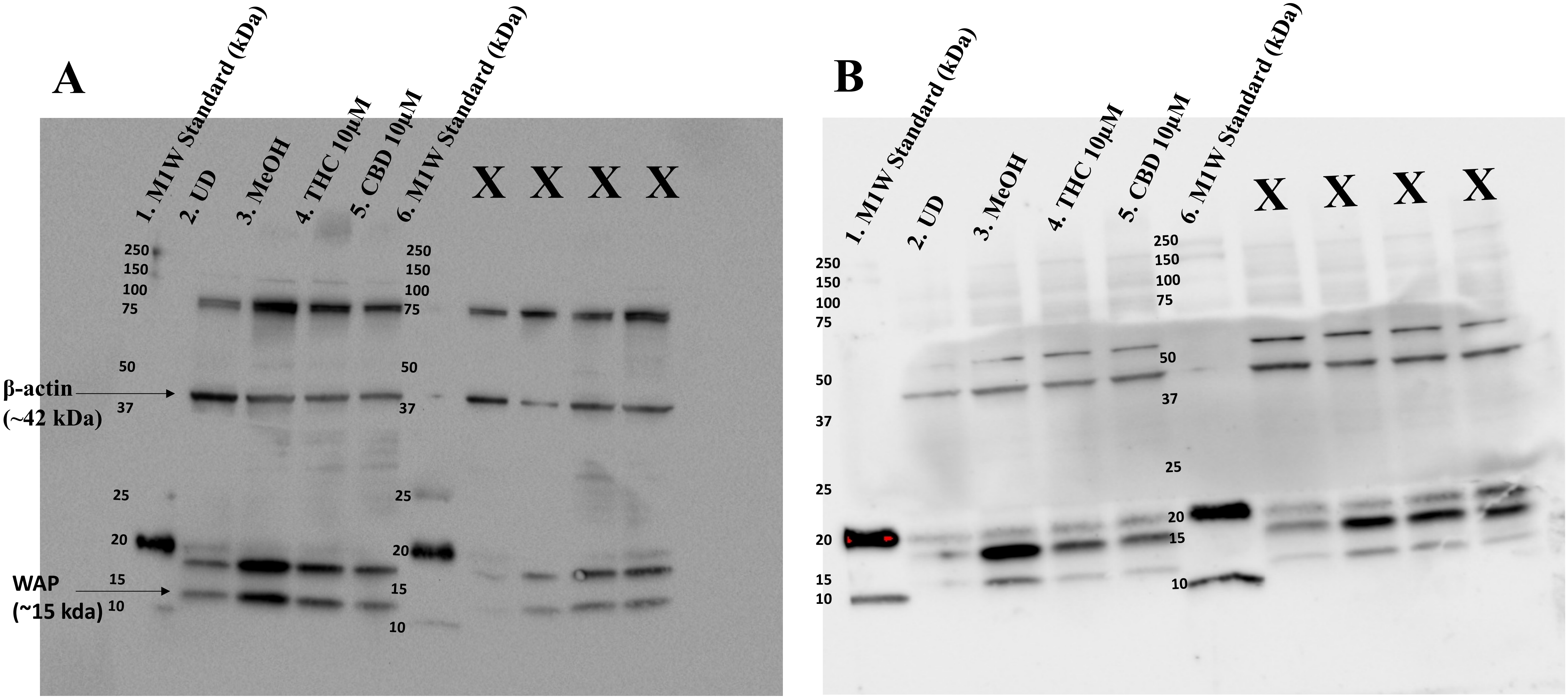

S1 Fig 3. 10µM CBD reduced WAP levels in HC11 cells, normalized to β-actin. HC11 cells were seeded in base media for 24 hours (UD), as described in the methods. HC11 cells were seeded in base media for 24 hours, supplemented with EGF and INS for 3 days, and differentiated in base media containing PRL, INS and DEX, as described in methods, and treated with vehicle control (MeOH), 10µM THC or 10µM CBD for 4 days, as described in methods. Cells from all conditions were processed for total protein isolation, as described in methods. For each sample, 15µg of total protein was separated on 12%, 10-well protein gel. The gel was transferred onto a PVDF membrane, blocked with 5% BSA for 2 hours at room temperature, blocked with 1:200 anti-WAP overnight at 4°C with gentle rocking. Next day, the PVDF membrane was incubated in 1:10000 anti-β-actin for 2 hours at room temperature, followed by incubation in 1:5000 of Goat Anti-Mouse IgG for 2 hours at room temperature. The proteins were detected by incubation of the PVDF membranes in ECL for 3 minutes and imaged using the Bio-Rad ChemiDoc^{TM} Imaging System. (A) Representative blot of WAP and β-actin expression in undifferentiated HC11 cells (UD), and cells treated with the vehicle control (MeOH), 10µM THC and 10µM CBD. Figure 4D was generated from lanes 2-5 in this data set. (B) Same membrane as (A), imaged at a different conditions, provided for clearer representation of the Molecular weight (MW) standards. Comparative analysis was performed between UD, MeOH, 10µM THC and 10µM CBD. Molecular weight (MW) standards have been labeled in lanes 1 and 6. Lanes 7-10 have been marked with an "X" as they were not included in the final figure reported in this study.

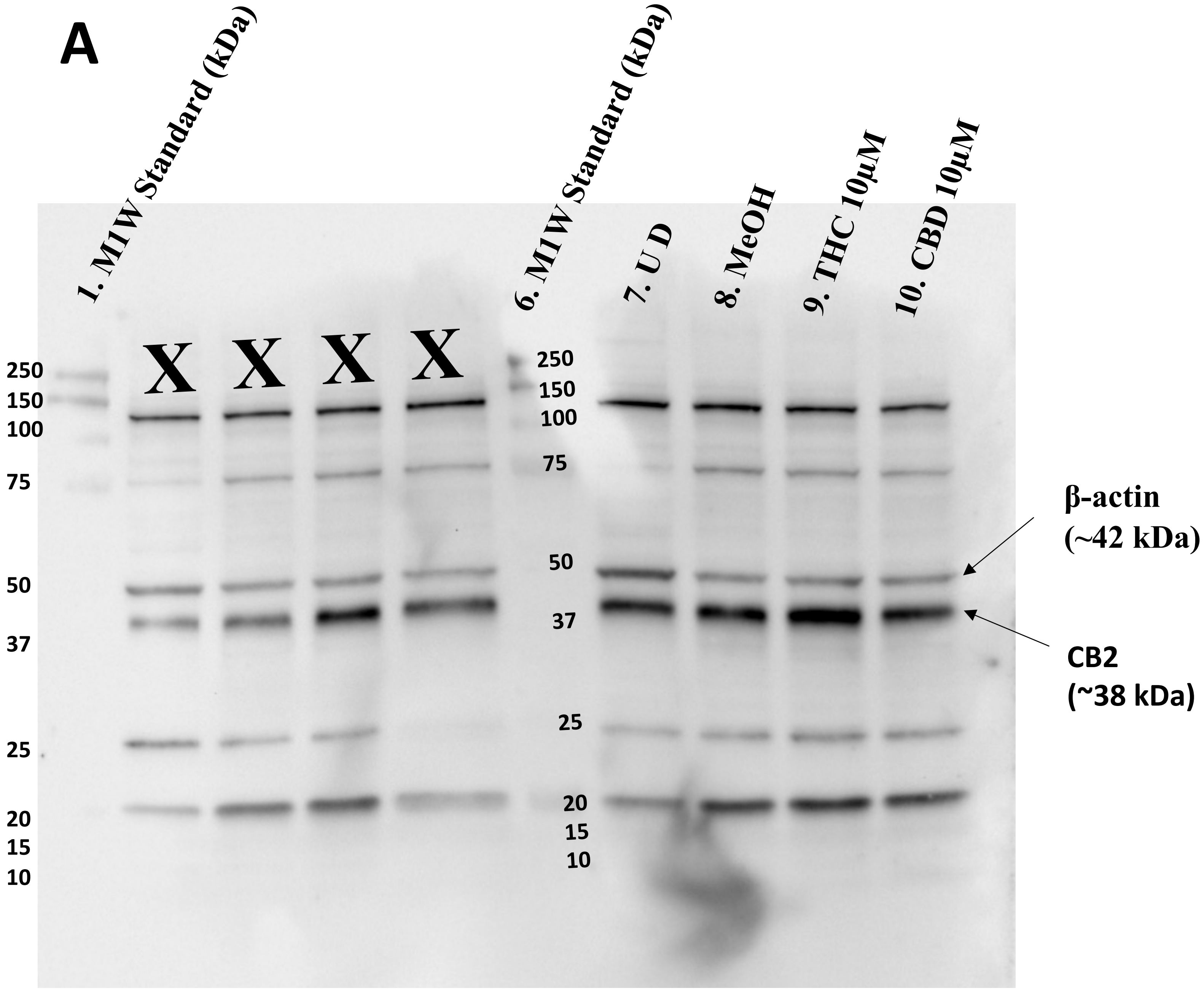

S1 Fig 4. 10µM CBD reduced CB2 levels in HC11 cells, normalized to β-actin. HC11 cells were seeded in base media for 24 hours (UD), as described in the methods. HC11 cells were seeded in base media for 24 hours, supplemented with EGF and INS for 3 days, and differentiated in base media containing PRL, INS and DEX, as described in methods, and treated with vehicle control (MeOH), 10µM THC or 10µM CBD for 4 days, as described in methods. Cells from all conditions were processed for total protein isolation, as described in methods. For each sample, 15µg of total protein was separated on 12% protein gel, 10-well protein gel. The gel was transferred onto a PVDF membrane, blocked with 5% BSA for 2 hours at room temperature, blocked with 1:200 anti-CB2 overnight at 4°C with gentle rocking. Next day, the PVDF membrane was incubated in 1:5000 of Donkey anti-rabbit IgG, for 2 hours at room temperature. Then, the PVDF membrane was incubated in 1:10000 anti-β-actin for 2 hours at room temperature, followed by incubation in 1:5000 of Goat Anti-Mouse IgG for 2 hours at room temperature, and imaged. (A) A representative blot of CB2 and β-actin expression in undifferentiated HC11 cells (UD), and cells treated with the vehicle control (MeOH), 10µM THC and 10µM CBD. Figure 6D was generated from lanes 7-10 in this data set. Comparative analysis was performed between UD, MeOH, 10µM THC and 10µM CBD. Molecular weight (MW) standards have been labeled in lanes 1 and 6. Lanes 2-5 have been marked with an “X” as they were not included in the final figure reported in this study.
